# Airway Epithelial Derived Cytokines and Chemokines and Their Role in the Immune Response to Respiratory Syncytial Virus Infection

**DOI:** 10.3390/pathogens8030106

**Published:** 2019-07-19

**Authors:** Lena Glaser, Patricia J. Coulter, Michael Shields, Olivier Touzelet, Ultan F. Power, Lindsay Broadbent

**Affiliations:** 1 Wellcome-Wolfson Institute for Experimental Medicine, School of Medicine, Dentistry and Biomedical Sciences, Queen’s University Belfast, Belfast BT9 7BL, Northern Ireland, UK; 2 Department of Paediatric Respiratory Medicine, Royal Belfast Hospital for Sick Children, Belfast BT12 6BE, Northern Ireland, UK

**Keywords:** respiratory syncytial virus, airway epithelium, innate immunity, inflammatory mediators, cytokines, chemokines

## Abstract

The airway epithelium is the primary target of respiratory syncytial virus infection. It is an important component of the antiviral immune response. It contributes to the recruitment and activation of innate immune cells from the periphery through the secretion of cytokines and chemokines. This paper provides a broad review of the cytokines and chemokines secreted from human airway epithelial cell models during respiratory syncytial virus (RSV) infection based on a comprehensive literature review. Epithelium-derived chemokines constitute most inflammatory mediators secreted from the epithelium during RSV infection. This suggests chemo-attraction of peripheral immune cells, such as monocytes, neutrophils, eosinophils, and natural killer cells as a key function of the epithelium. The reports of epithelium-derived cytokines are limited. Recent research has started to identify novel cytokines, the functions of which remain largely unknown in the wider context of the RSV immune response. It is argued that the correct choice of in vitro models used for investigations of epithelial immune functions during RSV infection could facilitate greater progress in this field.

## 1. Introduction

Respiratory syncytial virus (RSV) is a major respiratory pathogen and has been estimated to cause 64 million cases of respiratory disease each year [1]. Most RSV infections manifest as mild upper respiratory tract infections with symptoms such as cough, rhinorrhea, and coryza [2]. However, approximately one-third of children develop lower respiratory tract infections (LRTIs), and 2–3% progress to severe bronchiolitis and/or pneumonia requiring hospitalization [1]. Severe RSV LRTIs are characterized by extensive airway epithelial cell damage, as well as perivascular and peribronchiolar mononuclear cell infiltration [3,4] Despite the frequency of severe RSV LRTIs, little is known about the molecular mechanisms underpinning the disease. The evidence suggests that RSV infection does not induce the extensive epithelial cytopathology observed in severe LRTIs [5,6,7,8]. Instead, the observed tissue damage was hypothesized to be immune cell-mediated [6]. Paradoxically, immune responses to RSV infection have been portrayed as blunted and delayed when compared to other respiratory viruses, such as influenza [9,10]. Moreover, RSV infections have been linked to the development and exacerbations of childhood wheezing and asthma [11].

The airway epithelium is the primary target of RSV infection and has been implicated as a frontline defense system against respiratory viruses [12]. It acts as a physical barrier that prevents foreign material from accessing underlying tissues. Moreover, it is an immunological barrier that elicits inflammatory responses when appropriate [12]. Respiratory viruses, such as RSV, are known to modulate epithelial defenses, such as the physical barrier and ciliated cell function, sensor functions, and the secretion of inflammatory mediators to facilitate the infection of the airways. In vitro models that mimic the lung environment during RSV infections have started to elucidate the role of the airway epithelium in the control of cellular immunity associated with RSV infection. In this review, the authors have focused on studies demonstrating secretion of inflammatory mediators following RSV infection in vitro in correlation with in vivo data.

## 2. Methods of Methodological Review

A systematic search was conducted to identify English language articles published between 1990 and 2019 that discuss the secretion of inflammatory mediators from different airway epithelium models during RSV infection. 

The electronic databases SCOPUS, PubMed, and Google Scholar, were searched. The search terms investigated always included, cytokines, chemokines and alarmins as well as RSV, and one of the following terms for airway epithelial cell cultures: Airway epithelium, respiratory epithelial cells, airway epithelial cells, primary human airway epithelial cells, well-differentiated airway epithelial cells, paediatric airway epithelial cells, BEAS-2B, A549, Calu-3, 16HBE14o-, small airway Epithelial cells (SAE), nasal airway epithelial cells (NECs), pNECs, hPNECs, bronchial airway epithelial cells (BECs), pBECs, hBECs, tracheal airway epithelial cells and alveolar airway epithelial cells. There is a wide variety of terms used to describe primary airway epithelial cells in the literature. In this paper, the term, human airway epithelial cells (hAECs), is used to refer to all primary epithelial cell models discussed. 

The title and abstract of all publications were reviewed for eligible content before the full texts of the articles were reviewed to identify the cytokines and chemokines reported in the publication. The cell culture model was also listed. A table of all cytokines and chemokines was compiled and subsequently shortened based on the following inclusion criteria: Cytokines and chemokines reported by two or more independent publications; at least once in a primary cell model; and at least once by a protein-based method (Multiplex, ELISA) were included.

Inflammatory mediators that were previously reported to be associated with RSV infection but did not meet our inclusion criteria included: IL-7, IL-10, IL-12, IL-15, IL-16, IL-18, LIF, MIF, SCF, PDGF, M-CSF, TNF-β, IFN-β, CCL1 (I-309), CCL17 (TARC), CCL20 (MIP-3α, Exodus-1), CCL22 (MDC), CCL27 (CTAK), CXCL1 (GRO-α), CXCL2 (GRO-β), CXCL3 (GRO-γ), CXCL5 (ENA-78), CXCL-6 (GCP-2), CXCL16, CX_3_CL1 and Hsp-27. The exclusion of these cytokines and chemokines does not necessarily indicate an insignificant role during RSV infection. The secretion time-points (hours post-infection [hpi]) of the analytes listed in Table 1 result from experiments that were carried out under different conditions, which included various cell culture models, virus strains and multiplicities of infection (MOI).

## 3. Airway Epithelial Immune Response to RSV Infection

### 3.1. Epithelial Barrier Functions and Virus Infection

Epithelial integrity and the resulting barrier function of the airway epithelium are critical for a healthy lung environment. It is maintained by tight and adherens junctions, which form the apical junctional complex (AJC) in airway epithelial cells. They control the paracellular transport of environmental, viral, and microbial antigens from the lumen of the airways to the underlying tissue and immune cells [12]. The cilia, mucous and airway surface liquid (ASL) combine to form the mucociliary escalator which is essential in the clearance of pathogens and other inhaled foreign material [70]. RSV infection has a detrimental effect on ciliated cells, including cilia loss and mitochondrial damage [71]. Genetic polymorphisms that impair epithelial integrity have been associated with chronic respiratory disorders and severe disease in response to viral infections [72,73]. Interestingly, RSV has not been shown to reduce epithelial integrity. In fact, there is a consensus that the virus itself does not induce gross cytopathic effects in well-differentiated airway epithelial cells [6,7,8,74]. The lack of obvious cytopathology has been reinforced by findings that demonstrated an upregulation of junctional proteins, such as Claudin-4 and Occludin, following infection [42]. The expression of other junctional proteins, such as E-cadherin, ZO-1, and JAM-A, were unaffected by RSV infection [6,42]. These findings are consistent with the maintenance of airway epithelium integrity and polarity, indicating that the homeostatic physiological barrier defense of the epithelium and ciliary beat frequency are not dramatically impaired by RSV infection. However, the loss of ciliated cells, which can take ~14 weeks to normalize [75], is likely to reduce mucociliary clearance of pathogens.

### 3.2. Sensory Function of Epithelial Cells

In addition to acting as a physical barrier, airway epithelial cells act as innate immune sensors during infections. They express pattern recognition receptors (PRRs), such as Toll-like receptors (TLRs), RIG-I like receptors (RLRs), C-type lectin receptors (CLRs) and protease-activated receptors (PARs) [76]. It has been hypothesized that airway epithelial cells have a certain tolerance to microbial stimulation, which is guided by a reaction threshold that can be overcome in the context of infection [77]. The negative regulatory pathways of PRR signaling, as well as their localization on airway epithelial cells, have been proposed as mechanisms that influence the reaction threshold [78]. While this concept has mostly been explored in the context of bacterial infections, there are studies to suggest that similar mechanisms exist during RSV infection. TLRs have been suggested to contribute to antiviral immune responses in both in vivo and in vitro models, but their specific functions remain elusive [76]. Apical surface expression of TLR3, TLR4, and TLR7 in human tracheal sections and WD-hAEC cultures was previously reported [79]. Remarkably, the expression of TLR3 and TLR7 on the apical surface of airway epithelial cells is fundamentally distinct from the endosomal location of which they are commonly associated. This is interesting as it indicates possible interactions between viral particles and epithelial cells at the apical surface of epithelial cells. In this context, Monick et al. demonstrated that RSV infection increased the surface expression of TLR4 in monolayer hAEC cultures [80]. RSV F-protein is known to interact with TLR4 on airway epithelial cells [81]. Hence, TLR4 upregulation could represent a sensitization of epithelial cells to the virus. Similarly, the surface expression of TLR3 in monolayer hAEC cultures was shown to be upregulated during RSV infection [82]. Whether these observations translate to an increased apical expression on the surface in well-differentiated epithelium models remains to be investigated. Nevertheless, the interactions between RSV and TLRs have been implicated in the initiation of antiviral immune responses. The interactions between TLR3 and RSV have been implicated in the secretion of the cytokines CCL5 and CXCL10 [83]. Furthermore, interactions between RSV and TLR4 were recently implicated in the synthesis of type III interferons from the airway epithelium (Broadbent et al., unpublished observations). 

### 3.3. Epithelium-Derived Inflammatory Mediators during RSV Infection

The airway epithelium is the source of over 20 pro-inflammatory cytokines, chemokines, and growth factors during RSV infection (Table 1). The interactions between RSV and host airway epithelial cell PRRs have been suggested to induce the release of cytokines/chemokines [16,76,81] Alternatively, autocrine functions of cytokines and chemokines that are secreted early during RSV infection have been suggested to amplify and diversify the inflammatory mediators secreted [15,26]. CCL5, CXCL8, IL-6, and CXCL10 have been consistently reported as being secreted from the airway epithelium following RSV infection, as highlighted in several studies on nasopharyngeal secretions of children with RSV induced bronchiolitis [84,85,86,87,88]. Polymorphisms in the genes encoding the aforementioned cytokines have been shown to correlate with increased disease severity in infants [89,90,91]. The less well-characterized inflammatory mediators, such as tumor necrosis factor related apoptosis-inducing ligand (TRAIL), B-cell activating factor (BAFF), and thymic stromal lymphopoietin (TSLP) are implicated in the modulation of RSV-induced inflammatory responses (Table 1). TRAIL has been proposed to sensitize airway epithelial cells to apoptosis, which could contribute to a mechanism for the cell sloughing evident during RSV infection [40]. TSLP has recently become of interest in the context of the development of T_h_2-dominated immune responses to RSV infection [49], while BAFF was associated with the modulation of B-cell responses and the mechanisms that govern the generation of protective immunity in the mucosa [47]. Despite the suggested importance of these inflammatory mediators, the kinetics and mechanisms of their release from the airway epithelium and their specific role during RSV infection remain poorly understood. In addition, the epithelium secretes numerous growth factors, including G-CSF, GM-CSF, FGF, and VEGF, the functions and patterns of secretion of which are only starting to be explored in the context of RSV infection [32,34].

### 3.4. Secretion of Inflammatory Mediators

UV-inactivated virus particles were often used to determine if virus replication was needed to induce cytokine and chemokine release or if surface contact with viral proteins was sufficient. CCL5, CXCL8, IL-6, and CXCL10 were shown to be secreted in response to inactivated virus particles [16,28,64,66]. This indicated that the interaction of virus proteins with surface receptors triggers signaling cascades that lead to the release of inflammatory mediators. Indeed, evidence suggests that RSV proteins interact with PRRs on the surface of airway epithelial cells. Kurt-Jones et al. demonstrated interactions between the purified RSV F-protein and TLR4 and correlated this interaction to increased IL-6 secretion from monocytes stimulated with purified F-protein [81]. The study demonstrated this interaction on airway epithelial cells. However, an upregulation of TLR4 on the apical surfaces of epithelial cells was previously reported during RSV infection, indicating that airway epithelial cells can interact directly with the virus during infection [80]. Moreover, Oshansky et al. demonstrated that IL-1α, CCL5, CXCL8, and CXCL10 were secreted in response to both UV-inactivated virus as well as purified RSV G and F-proteins [16]. These results are consistent with the idea that interactions between RSV proteins and the apical epithelial surface initiate secretion of cytokines and chemokines during infection. The specific signaling pathways implicated in the release of these inflammatory mediators during RSV infection remain incompletely understood.

### 3.5. Autocrine Signaling Amplifies Inflammatory Mediator Release

**Figure 1:**
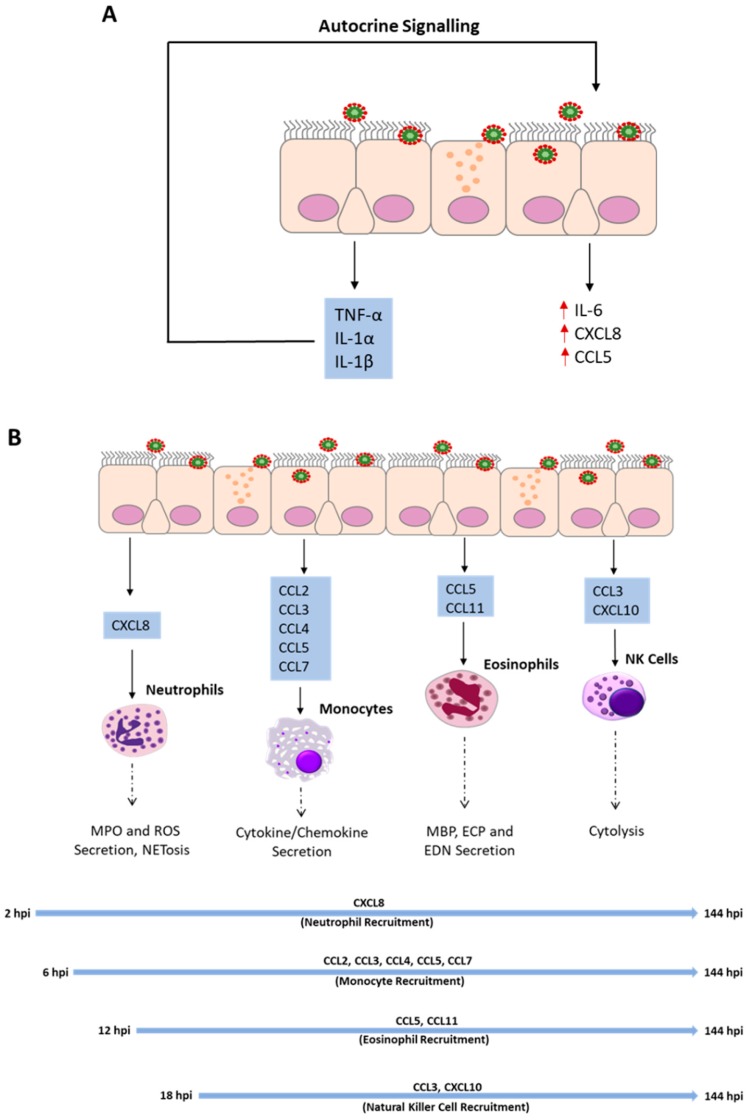
Autocrine signaling of TNF-α, IL-1α, and IL-1β amplifies the secretion of the proinflammatory cytokine IL-6 and the chemokines CXCL8 and CCL5 following RSV infection (**A**); Primary airway epithelial cells and airway epithelial cell lines secrete chemokines at different time points throughout the course of RSV infection as outlined in Table 1. The chemokines facilitate the recruitment of innate immune cells from the periphery to the lung where they implement antiviral actions through various cell-specific mechanisms (**B**). Major Basic Protein (MBP), Eosinophil Cationic Protein (ECP), Eosinophil-derived neurotoxin (EDN), Myeloperoxidase (MPO), reactive oxygen species (ROS).

Cytokines and chemokines secreted during RSV infection can be divided into primary cytokines that are directly induced by virus infection, and secondary cytokines induced by other inflammatory mediators. The secretion of IL-6 and CXCL8 are upregulated as a consequence of autocrine actions of TNF-α, IL-1α, and IL-1β following RSV infection [14,15,26]. The addition of neutralizing antibodies targeting IL1-α, IL-1β, and TNF-α, individually, or combined, during RSV infection was shown to significantly decrease the secretion of IL-6 and CXCL8 [15,26]. Furthermore, the secretion of TNF-α from RSV infected airway epithelial cells was shown to increase the secretion of CCL5 [30]. A closer analysis of TNF-α, IL-1α, and IL-1β identified IL-1α as the most potent inducer of IL-6 and CXCL8 [15,26]. Interestingly, TNF-α was shown to have an inhibitory effect on the secretion of CXCL8 in the first 24 h post-infection (hpi). However, at 48 hpi, it amplified the secretion of CXCL8 [15]. Coinciding with this, a biphasic release of CXCL8 had previously been reported [66] and was eventually suggested to be associated with the autocrine effect of TNF-α [64]. IL-1α and TNF-α amplify the release of inflammatory mediators from the airway epithelium by autocrine effects, as shown in Figure 1A. Whether other cytokines possess the same ability remains to be explored.

### 3.6. IFNs Amplify Cytokine and Chemokine Release from Epithelial Cells 

Type III interferons, and specifically IL-29/IFNλ1, were shown to be the principle interferon secreted from airway epithelial cells during RSV infection [6,7,44,45]. However, the mechanisms of induction, secretion kinetics of IL-29 following RSV infection, and its downstream effects, remain incompletely understood. Recent work from our group indicated that the release might be TLR4-dependent (Broadbent et al., unpublished observations), while downstream effects of IL-29 signaling included Mx protein responses [44]. Type I interferons (IFNα/β) are another class of cytokines that induce inflammatory mediators. However, reports of the secretion of type I IFNs from in vitro models of RSV-infected epithelial cells are limited. There are no reports of IFNα secretion from the airway epithelium and few reporting the secretion of IFN-β, suggesting that they are unlikely to be the primary drivers of inflammatory mediator secretion. Interestingly, recent data from mouse models suggested that alveolar macrophages (AM) might be the major source of type I IFNs following RSV infection [92]. However, Schijf et al. demonstrated a significant secretion of IFN-β from A549 cell lines in vitro [27]. Other studies investigating the secretion of INF-β from cell lines have shown this to be cell line-specific and dependent on a high multiplicity infection [13,45]. Importantly, research in differentiated primary airway epithelial cell cultures indicated that type I IFN responses were limited, at best, in response to RSV infection [6,7,44]. Nevertheless, it was recently shown in mouse models that the absence of IFNARI during RSV infection reduced the number of inflammatory mediators found in the lungs of infected animals. This indicates that IFNα/β influence airway epithelial cells, amongst others, in a paracrine fashion [93]. The interactions between airway epithelial cells and other tissue resident immune cells, such as dendritic cells and interstitial macrophages, have also been suggested to contribute to the inflammatory environment during RSV infection. However, the functions of those cells are beyond the scope of this article and have been reviewed extensively elsewhere [94,95].

### 3.7. Peripheral Blood Cell and Epithelial Cell Interactions

Table 1 demonstrates that chemokines constitute most of the known inflammatory mediators secreted from airway epithelial cells following RSV infection. This suggests that the airway epithelium plays an important part in the recruitment of immune cells from the periphery. CXCL8, CCL5, and CXCL10 are well-known chemo-attractants for neutrophils, eosinophils and natural killer cells (NKs), which have been proposed to represent the first wave of innate immune cells recruited to the lungs during RSV infection, as shown in Figure 1B [96]. In addition, less well characterized epithelial-derived chemokines, such as CCL11, which promote eosinophil recruitment, have been identified [64]. Furthermore, epithelial-derived CCL2, CCL3, and CCL4 are important for the efficient recruitment of monocytes and natural killer cells during RSV infection [55,59,97]. Interestingly, significant numbers of neutrophils and eosinophils have been found in the lumen of the airways during RSV infection [3,98], indicating that they migrate through the epithelium. The upregulation of adhesion molecules, such as intercellular adhesion molecule 1 (ICAM-1) and vascular cell adhesion protein 1 (VCAM-1), in monolayer airway epithelial cell models have been suggested to promote transepithelial migration of innate immune cells during influenza virus infection [99]. Similarly, the upregulation of ICAM-1 and VCAM-1 in monolayer epithelial cell models were demonstrated following RSV infection [100]. However, to the best of the authors’ knowledge, there are no studies in WD-hAECs demonstrating a uniform upregulation of these receptors across all layers of the airway epithelium following RSV infection. Thus, the role of ICAM-1 and VCAM-1 in transepithelial migration remains unclear. Furthermore, there are studies that indicated a polarized release of epithelium-derived cytokines during RSV infection [9,16,64]. Two of these studies reported a predominantly basolateral release of chemokines, such as CXCL8, CCL5, and CXCL10 [9,64]. In contrast, Oshansky et al. reported a predominantly apical release of IL-1α [16]. Recent unpublished work from our group indicated that the secretome of well-differentiated airway epithelial cell cultures is distinct at the apical and basolateral surfaces. This profile is altered during RSV infection (Touzelet et al. unpublished observations). The significance of a polarized release of proteins from airway epithelial cells remains to be explored in detail. However, it supports a hypothesis for the establishment of a transepithelial chemotactic gradient that facilitates the migration of inflammatory cells into the lumen of the airways during RSV infection.

### 3.8. Differential Secretion of Cytokines/Chemokines Following Infection with Common Respiratory Viruses

Respiratory viruses induce similar but not identical responses following the infection of airway epithelial cells. For example, Ioannidis et al. demonstrated secretion of IL-6, CXCL8, CXCL9, CXCL10, CXCL11, G-CSF and GM-CSF into the basolateral compartment of both RSV and influenza virus infected cultures [9]. However, the paper highlighted that despite similarities, the two viruses induced qualitatively and quantitatively distinct responses in airway epithelial cells. Interestingly, influenza elicited a more robust response characterized by the secretion of more diverse inflammatory mediators compared to RSV [9]. Human metapneumovirus (hMPV), which is closely related to RSV, has been associated with the release of IL-6,CXCL8,CXCL10, RANTES, CCL2, CCL3 and CCL20 from airway epithelial cells following infection in vitro [101]. This closely resembles the major chemokines secreted from airway epithelial cells following RSV infection in vitro (Table 1). Nevertheless, mouse studies and analyses of nasal washes from children with hMPV and RSV infections demonstrated distinct responses elicited by the viruses [102,103]. hMPV resulted in secretion of lower levels of cytokines, such as IL-6, IL-1 and TNF-α, when compared to RSV [102,103]. Moreover, it appears that hMPV induces higher levels of GM-CSF and more potent type I IFN responses compared to RSV in mice [104]. The analyses of nasopharyngeal aspirates of children with hMPV infection, however demonstrated a dominant IL-28 and hence type III IFN response [104]. In addition, IL-18 was reported to be secreted following hMPV infection [104]. While a type III IFN dominated immune response would be expected in RSV infection, the secretion of IL-18 has not been explored in much detail in the context of RSV infection. In vitro airway epithelial cell line models of rhinovirus infection detail an increased secretion of IL-6, IL-15, CXCL1, CXCL5, CXCL8, CXCL10, HGF and TNF-α amongst others [105,106,107]. The presence of IL-15, CXCL1 and HGF is notable as these mediators are not commonly reported as being secreted from airway epithelial cells following RSV infection (Table 1). The differences in airway epithelial immune responses elicited by RSV and other respiratory viruses could account for differential recruitment of leukocytes.

## 4. Immune Cell Recruitment to the Airway Epithelium

A considerable amount of knowledge about the immune response to RSV infection in human cells/tissues derives from clinical studies. The analysis of bronchoalveolar lavage (BAL) and nasal lavage (NAL) fluids, as well as peripheral blood samples from infected individuals, has allowed the identification of critical cytokines, chemokines and immune cells involved in RSV immunity. Neutrophils, eosinophils, monocytes, and NK cells were identified as the main peripheral immune cells recruited to the lung following RSV infection. Figure 1B outlines the key epithelial-derived chemokines involved in the recruitment of leukocytes from the periphery at different time points throughout the course of the infection, and their proposed antiviral activities, the importance of which is discussed below.

### 4.1. Neutrophils

Neutrophils are the most common cell type found in the lumen of the airways during RSV infection in infants [88,98,108]. It is not yet clear whether neutrophils are beneficial or detrimental in RSV infection, but it was hypothesized that they contributed to both viral clearance and tissue damage [109,110]. CXCL1-8 are the main chemokines associated with neutrophil trafficking [58,111]. CXCL8 is the best-known epithelial-derived neutrophil chemoattractant in RSV infection (Table 1). The analysis of BAL fluids identified neutrophils as a major cell population in the lumen of the airways of children with severe RSV bronchiolitis [98,108], indicating that the cells migrate across the epithelium. The transepithelial migration was suggested to be accomplished via ICAM-1 expression on epithelial cells and its interaction with CD11a and CD11b on the surface of neutrophils [112,113]. 

It is not clear if the antiviral activity of neutrophils relies on the destruction of infected cells or direct interactions with the virus. The activation of neutrophils has been shown to depend on the presence of cytokines and chemokines and results in degranulation, oxidative bursts, inflammatory mediator release, and NETosis [114]. Histopathology reports of autopsy tissues from children that succumbed to RSV bronchiolitis demonstrated extensive tissue damage in the bronchioles [3,115]. The functions of activated neutrophils were implicated in the generation of cytopathology following RSV infection [4,110]. This hypothesis is consistent with a recent study by Deng et al., which demonstrated enhanced cytopathology in co-cultures of infected epithelial cells and neutrophils [113]. The study does not identify a specific mechanism causing the tissue damage. However, it emphasizes the expression of myeloperoxidase (MPO) on the surface of transmigrated neutrophils. The presence of MPO has previously been reported on the DNA fibers of NETs formed in response to RSV exposure [116]. The antiviral effects of MPO have not specifically been investigated in the context of RSV infection, but NET-bound MPO was shown to inactivate HIV-1 [117]. In addition to MPO, neutrophil elastase was implicated in contributing to RSV pathology. Elevated concentrations of the enzyme were identified in BAL fluids of infants with acute bronchiolitis [118], and the proteolytic effects of the enzyme have been implicated in the induction of tissue damage [110]. Moreover, in vitro studies demonstrated NET fibers decorated with neutrophil elastase during RSV infection [116]. However, specific antiviral activities of neutrophil elastase in RSV infection have not yet been shown. Another mechanism through which neutrophils are thought to mediate antiviral effects is the secretion of reactive oxygen species (ROS). It has been shown that the inflammatory environment during RSV infection promoted the induction of oxidative bursts [114]. However, the direct effect of ROS secretion from neutrophils on RSV inactivation has not yet been investigated.

Neutrophils cultured in NAL fluid from infants with RSV bronchiolitis displayed an enhanced survival compared to those cultured in NAL fluids from uninfected controls [119]. This phenomenon was attributed to inflammatory molecules that delayed neutrophil apoptosis during RSV infection, and cytokines, such as IL-6, G-CSF, IFN-γ, and TNF-α, were implicated in this process [120,121]. In addition, a recent study by Coleman et al. demonstrated that a still unknown monocyte-derived soluble mediator prevented neutrophil apoptosis during RSV infection, suggesting a possible synergy between the cells [122].

### 4.2. Monocytes

Monocytes were found in the lung interstitium in fatal cases of RSV bronchiolitis [3]. They have been implicated as facilitator cells during RSV infection, supporting the proliferation, activation, and survival of other immune cells. Moreover, they are thought to be the precursors of differentiated macrophages and dendritic cells in the inflamed tissues [123].

Recent evidence suggests that monocytes may be primed in the peripheral blood before entering the airways. Ahout et al. analyzed peripheral blood samples from infants diagnosed with RSV bronchiolitis and demonstrated an increased number of intermediate phenotype monocytes (CD14^+^/CD16^+^) compared to healthy controls [124]. This potentially reflects an immune activation that facilitates the subsequent recruitment of monocytes to the inflamed airways. The upregulated expression of the chemokine receptors CCR1, CCR2 and CCR5 in peripheral blood monocytes treated with conditioned medium from RSV-infected airway epithelial cells indicated that airway epithelial cells are involved in the recruitment of these cells [125]. Epithelium-derived CCL2, CCL3, CCL4, CCL5, and CCL7 were previously implicated in mediating monocyte chemotaxis [59]. Interestingly, it was shown that the absence of AM-derived type I IFNs significantly impaired the recruitment of monocytes to infected airways in mice. This was attributed to the absence of type I IFN-induced CCL2 secretion [92,93]. The presence of CCL2 in BAL fluids of infants with RSV-induced bronchiolitis indicates an important role for this chemokine in vivo [119,120,126,127]. However, it remains to be shown whether type I IFN amplifies CCL2 secretion from airway epithelial cells in vitro. Table 1 demonstrates that numerous studies reported the secretion of CCL2 from epithelial cells in the absence of detectable type I IFNs, indicating that their secretion from epithelial cells is independent of IFN-α/β.

The absence of monocytes delayed viral clearance in mice, but did not hinder the eventual resolution of the disease, as shown by their capacity to regain weight after infection [92]. This indicates that monocytes contribute to disease resolution, but are not essential. Thus, the antiviral functions of monocytes remain elusive. Early studies indicated that monocytes reduced the spread of the virus to adjacent, uninfected cells [128]. However, the mechanisms were not explored. Moreover, it was suggested that inflammatory monocytes recruited to the lungs differentiate into DCs and macrophages [123]. However, the authors are not aware of studies that specifically explored the fate of inflammatory monocytes after their recruitment to the lungs following RSV infection.

The secretion of cytokines and chemokines from monocytes has been implicated as a major function of the cells during RSV infection [123]. Recent evidence demonstrated that the airway epithelium can influence inflammatory mediator release from monocytes via the release of high-mobility group box 1 (HMGB1) [36]. HMGB1 is an alarmin that is secreted from cells in the context of inflammation and infection [69]. Moreover, it also induces the release of cytokines and chemokines, such as IL-1β, IL-1RA, IL-6, CXCL8, IL-10, TNF-α, G-CSF, IFN-γ, CXCL10, CCL2, CCL3, CCL4 and CCL5 from monocytes [36]. Nasal aspirates from children with severe bronchiolitis consistently contain these cytokines suggesting an important role for them in RSV infection [84,85,86,88]. However, whether monocytes are a principal source of these inflammatory mediators in BAL fluids of infants with severe RSV bronchiolitis remains to be explored.

### 4.3. Eosinophils

The BAL of children with RSV induced bronchiolitis demonstrated that eosinophils constitute a minority cell population of the luminal infiltrate during RSV infection [98]. This finding coincides with histological studies on the airways of children that succumbed to RSV bronchiolitis [3,115]. Eosinophils are thought to be recruited to the lungs by epithelium-derived chemokines, such as CCL3, CCL5, and CCL11 [59,60,65,117].

The detection of eosinophil-specific degranulation products, such as eosinophil cationic protein (ECP) and eosinophil-derived neurotoxin (EDN), in BAL and NAL fluids of infants with severe RSV indicates the activation of the cells following RSV infection [60,129,130]. This was confirmed in vitro, where the production of superoxide from eosinophils after exposure to RSV indicated their activation in response to direct contact with the virus [131]. Moreover, ECP and EDN reduced the infectivity of RSV after direct exposure, suggesting that they have antiviral activities [132,133,134]. Furthermore, a recent study identified major basic protein (MBP) as another eosinophil-derived antiviral protein and reported that MBP promotes the cell death of RSV-infected epithelial cells [38]. Despite these findings, it is not clear whether eosinophils are beneficial or detrimental in the resolution of viral infections. The evidence suggests that RSV infections stimulate T_h_2-dominated immune responses [11]. In fact, high serum levels of RSV-specific IgE in infants with bronchiolitis correlate with increased disease severity and eosinophil counts and are associated with the development of wheeze and asthma in childhood [135,136]. Interestingly, patients with RSV bronchiolitis are much more likely to be hospitalized, and have a longer hospital stay, if there is a family history of atopy [137,138]. Similarly, there is correlation between atopy and asthma development [139]. However, the precise relationship between RSV infection, atopy and the onset of childhood wheeze and asthma remains to be elucidated.

### 4.4. Natural Killer Cells

Natural killer (NK) cells are innate immune lymphocytes recruited to the lungs during RSV infection. It was previously shown that the numbers of NK cells in the peripheral blood were reduced in infants with severe RSV infection and that this correlated with increased disease severity [140]. The reduced NK cell numbers in the peripheral blood likely reflect the increased recruitment of the cells to the lung parenchyma. This was confirmed in mouse studies, which demonstrated an accumulation of NK cells in the lung after infection [141,142]. Surprisingly, an attenuation of lung injury was observed once NK cells were depleted in mice [142]. However, the role of NK cells in RSV infection remains poorly understood. NK cells likely contribute to viral clearance through the release of cytotoxic granules and the formation of synapses that induce cytolysis. Furthermore, the secretion of cytokines from NK cells, specifically IFN-γ, was suggested to promote disease resolution [143]. 

The activation of NK cells depends on the formation of receptor-mediated synapses [143]. Interestingly, RSV infection leads to an upregulation of stressed-induced ligand MHC class I polypeptide-related sequence A (MICA) on BEAS-2B cells [144]. MICA was previously associated with the activation of NK cells that express the cytotoxicity receptor NKG2D [145]. This suggests that epithelial cells are primed for NK cell-mediated cytolysis. However, it was also shown that RSV-infected epithelial cells upregulated the expression of MHCI and soluble MICA, both of which are known inhibitors of NK cell cytotoxicity [144,146]. Both of these findings suggest that NK cell-mediated cytolysis is tightly controlled during RSV infection. In the context of these findings, Zdrenghea et al. hypothesized that an initial upregulation of MICA in infected airway epithelial cells lead to the activation of NKG2D-expressing NK cells. These activated NK cells subsequently serve as a source of IFN-γ, which initiates a negative feedback loop for the expression of MICA on epithelial cell surfaces. Consequently, NK cell-mediated cytolysis is inhibited. Furthermore, IFN-γ has been associated with an increased expression of IL-15 from infected airway epithelial cells [144]. IL-15 has been associated with increased survival and activation of CD8^+^ T-cells. Thus, it may be hypothesized that the release of IFN-γ from NK cells promotes a switch to antigen-specific cytotoxicity mediated by CD8^+^ T-cells. 

## 5. Models of RSV Infection

A variety of models that mimic RSV infection in humans are used to study RSV pathogenesis. The ethical and practical considerations hinder investigations of the disease in human subjects. Consequently, animal models are widely used as surrogates. Small animal models reproduce some aspects of human RSV LRTIs, but they cannot replicate the full disease spectrum of human RSV disease [147]. The evidence suggests that chimpanzees, as natural hosts of the virus, suffer clinical consequences of RSV infection that closely resemble those seen in humans [148,149]. However, as a protected species, research is no longer permissible in chimpanzees. Recently, human challenge models have emerged as a novel way to investigate the consequences of RSV infections [150]. The use of healthy adults as test subjects is a limitation of these studies. Adults have a substantially developed immunity to RSV and do not represent a risk group for severe RSV disease. Accordingly, the clinical consequences of RSV challenges are much more limited when compared to RSV-naïve infants. However, the recruitment of infants to conduct pediatric studies is ethically impossible.

The in vitro models allow for more specific analyses of the immunological role of the airway epithelium during RSV infection. The immortalized cell lines, such as the A549 (type II alveolar epithelial carcinoma cell line) and BEAS-2B (immortalised bronchial epithelial cell line) are most frequently used to study RSV infection in vitro (Table 1). Both cell lines reproduce some characteristic hallmarks of RSV infection in vivo. BEAS-2B cells, for example, restricted RSV infection to small foci [13]. This pattern was previously reported from histopathological studies on the airways of fatal cases of RSV disease [3,115]. Furthermore, BEAS-2B cells generate characteristic antiviral immune responses, such as the expression of interferon-stimulated genes and PRRs [13]. Interestingly, Fonceca et al. demonstrated increased patterns of RSV infection, replication, and cytotoxicity in primary airway epithelial cell monolayers compared to BEAS-2B cells [23]. This suggests that BEAS-2B cells have a more pronounced, intrinsic antiviral state compared to primary cells. In contrast, A549 cells are readily susceptible to RSV infection and do not exhibit the characteristic infection pattern seen in histopathological studies [13]. They generate strong pro-inflammatory responses, characterized by the release of cytokines and chemokines upon infection with RSV [13]. The increased secretion of CXCL8, CCL5, and CCL2 indicates that this response would favor the recruitment of innate immune cells, such as neutrophils and eosinophils, which are associated with the induction of the epithelium cytopathology that is characteristic of severe RSV infections [3,115]. Interestingly, it was shown that the secretion of cytokines/chemokines and the expression of PRRs in A549 cells following RSV infection was similar, but not identical, to the responses of primary epithelial cells [17]. It is not currently understood if the different response patterns seen between cell lines and primary epithelial cells are a consequence of the immortalization process, polarization, differentiation or because they originate from different regions of the bronchial tree.

The ability to take samples from routine bronchoscopies has made primary airway epithelial cells more accessible for research [151]. The authors and other researchers have shown that nasal airway epithelial cells are reliable surrogates for bronchial epithelial cells in terms of RSV cytopathogenesis [6,152,153]. Due to the ease of access, this important development allows the procurement of airway epithelial cells from diverse patient cohorts, thereby massively expanding the capacity to study RSV pathogenesis in humans. Many studies use primary cells in submerged monolayer cultures. However, recent evidence indicated that polarized well-differentiated primary airway epithelial cells (WD-PAECs) represented the most morphologically and physiologically relevant model of the airway epithelium in vitro [6,7,8]. Polarization is a crucial feature of WD-PAECs as it reflects the specific, polarized expression of PRRs seen in vivo [79]. Despite the countless benefits of this experimental platform, there are disadvantages. The need for trained clinical staff to obtain the cells, as well as more costly culturing methods and low cell yields, are limiting factors [151]. Moreover, the behavior of cultured primary airway epithelial cells may still vary from cells in vivo due to the absence of surrounding cells and tissues and associated cross-talk. This issue is being addressed through the development of co-culture models, most notably the development of the lung-on-a-chip system [154].

## 6. Conclusions

This paper reviewed the importance of the airway epithelium in the control of innate immune responses to RSV infection, with a particular emphasis on the recruitment of peripheral immune cells. The secretion of more than 20 inflammatory mediators during RSV infection indicated that the airway epithelium contributes to the control of the local immune response. The high level of chemokine secretion reportedly released from the airway epithelium suggests a key role in the recruitment and activation of peripheral immune cells. Despite this, only a few in vitro studies have investigated the role of epithelium-derived cytokines in the context of RSV infection in the past two decades. The exclusion criteria in this review did not permit the addition of all reported cytokines and chemokines into Table 1 due to the low frequency of reports testing for their presence. The recent progress in protein quantification and the development of new RSV infection study models will facilitate an expansion of our knowledge in this field. It is increasingly clear that the airway epithelium is an important component of the immune response to RSV infection. Thus, a better understanding of the role the airway epithelium plays in early immune responses is critical to the future development of pharmaceutical solutions to RSV disease.

## Figures and Tables

**Table 1:** A list of inflammatory mediators derived from the airway epithelial models during RSV infection, their roles in inflammation, the time-points at which they were found to be secreted and the cells they are known to influence.

Name	Cell Culture Model	Known Functions in Inflammation	Interactions with Other Cells	Secretion Time Point (hpi)
**IL-1α/FAF**	WD-hAECs, A549, SAEs, A549, and BEAS-2B [9,13,14,15,16,17]	Pleiotropic cytokine; initiates self-perpetuating inflammatory responses; known pyrogen; induces hyperalgesia and vasoconstriction [18]	Induces the secretion of cytokines and chemokines from tissue cells and lymphocytes via IL-1R1 signaling [18]	12 [16] 24 [17] 48 [9,13,14,15]
**IL-1β**	A549 and SAEs [14,15,17]	Pleiotropic cytokine, initiates self-perpetuating inflammatory responses; known pyrogen; induces hyperalgesia and vasodilation [19]	Induces the secretion of cytokines and chemokines from tissue cells and lymphocytes via IL-1R1 signaling [19]	24 [17] 48 [14,15]
**IL-6**	WD-HAECs, A549, SAEs, BEAS-2B, hAECs [6,7,9,13,17,20,21,22,23,24,25,26,27,28,29,30]	Pleiotropic cytokine; induces acute phase protein secretion, influences T- and B-cell growth and maturation [31]	Induces the secretion of inflammatory mediators from tissue and immune cells via IL6R signaling [31]	6 [28] 12 [23] 24 [6,7,17,26,27,28,29] 48 [9,13,26,30] 72 [24] 96 [6,7,20,21,22] 120 [7] 144 [7]
**VEGF**	A549, SAEs, hAECs [17,32]	Angiogenesis and vascular remodelling [33]	Activates endothelial cells [33]	6 [32] 24 [17,32] 48 [32]
**FGF**	A549 and SAEs [17,34]	Pleiotropic growth factor involved in tissue repair and regeneration; associated with cell proliferation and angiogenesis [35]	Activates endothelial cells and tissue cells expressing Fibroblast growth factor receptor (FGFR) to induce angiogenesis and proliferation [35]	24 [17,34]
**G-CSF**	WD-hAECs, A549, and SAEs [9,17,36]	Immunostimulation of neutrophils; immunosuppression of myelomonocytic cells [37]	Regulates neutrophil development and survival; modifies cytokine production from neutrophils, monocytes, macrophages, and DCs [37]	24 [17,36] 48 [9]
**GM-CSF**	WD-hAECs and A549 [17,20,22,38]	Immunostimulation of myelomonocytic cells [39]	Activates all myelomonocytic cells that express the GM-CSF receptor and promotes their survival and differentiation [39]	24 [17] 48 [38] 96 [20,22]
**TRAIL**	WD-hAECs, A549, and SAEs [6,7,17,40]	Induces apoptosis in virus-infected cells and tumor cells; implicated in the regulation of T-cell homeostasis [41]	Interact with infected or tumorigenic tissue cells that express TRAIL receptors DR4 and DR5, influences the expansion and maturation of CD4^+^ and CD8^+^ T-cells via DR4 and DR5 signaling [41]	24 [17] 96 [6,7] 120 [7] 144 [7]
**TNF-α**	WD-hAECs, A549, and SAEs [9,14,15,17,27,42]	Pleiotropic cytokine; promotes leukocyte extravasation; known pyrogen, promotes vasodilation, involved in the regulation of the coagulation cascade [43]	Promotes inflammation by interacting with TNFR1 expressing cells, especially known for the activation of endothelial cells [43]	24 [17,27,42] 48 [9,14,15,42] 72 [42]
**IFNλ/IL-29**	WD-hAECs, hAECs, A549 and BEAS-2B [6,13,44,45]	Stimulate innate antiviral mechanisms [46]	Thought to mostly act on epithelial cells in various organs [46]	24 [45] 48 [13] 72 [13] 96 [6,13,44]
**BAFF/TNFSF13B**	WD-hAECs, hAECs [9,47]	Regulates B-cell homeostasis, promotes the induction of pathogen specific antibody production [48]	Interacts with B-cells via BAFF receptor (BAFF-R) or transmembrane activator and calcium-modulating cyclophilin ligand interactor (TACI) signalling [48]	48 [9,47]
**TSLP**	hAECs [49,50]	Stimulates haematopoietic cells to induce T_h_2 responses; associated with blockage of T_h_1 and T_h_17 responses [51]	Interacts with Monocytes, DCs, CD4^+^ T-cells, B-cells and eosinophils via TSLP receptor (TSLP-R) [51]	24 [49]
**TGF-β**	WD-hAECs, hAECs, A549 and BEAS-2B [52,53]	Pleiotropic cytokine; regulates peripheral tolerance; regulates T-cell homeostasis and survival; promotes T_h_17 cell differentiation; suppresses cells of the innate immune system [54]	Interacts with tissue cells and leukocytes that express the TGF-β receptors TGF-β I and II [54]	24 [52]
**CCL2/MCP-1**	WD-hAECs, hAECs A549, SAEs, BEAS-2B [9,13,16,17,55,56,57]	Mainly involved in monocyte trafficking [58]	Recruits monocytes via CCR2 and CCR4 signaling [58]	6 [16] 24 [16,17,56] 48 [9,13,55]
**CCL3/MIP-1α**	WD-hAECs, hAECs A549, SAEs, BEAS-2B and Hep-2 [9,17,25,55,57,59,60]	Mainly promotes monocyte and NK cell trafficking [58]	Recruits monocytes, macrophages and NK cells via CCR1, CCR4 and CCR5 signaling [58]	24 [17] 48 [9,55,59] 72 [60] 96 [60] 120 [60]
**CCL4/MIP-1β**	WD-hAECs, hAECs, A459 and SAEs [9,17,25,57]	Mainly promotes monocyte and NK cell trafficking [58]	Recruits monocytes, macrophages and NK cells via CCR1, CCR5, and CCR8 signaling [58]	24 [17] 48 [9]
**CCL5/RANTES**	WD-hAECs, hAECs, A549 and SAEs, HEp-2 [6,7,9,13,16,17,21,24,25,29,30,36,45,55,56,57,59,60,61,62,63,64,65]	Mainly promotes monocyte and NK cell trafficking [58]	Recruits monocytes, NK cells, basophils, DCs and eosinophils via CCR1, CCR3, CCR4 and CCR5 signaling [58]	12 [45,61] 24 [6,17,24,29,36,45,56,61,62,63] 48 [9,13,24,30,55,59,61,63,64] 72 [24,63] 96 [6,7,21,60] 120 [7] 144 [7]
**CCL7/MCP-3**	WD-hAECs and SAEs [9,17]	Mainly involved in monocyte recruitment [58]	Recruits monocytes via CCR1, CCR2 and CCR3 signaling [58]	24 [17] 48 [9]
**CCL11/Eotaxin**	WD-hAECs, hAECs [17,65]	Promotes eosinophil and basophil recruitment [58]	Recruits eosinophils, basophils via CCR3 signaling [58]	24 [17]
**CXCL8/IL-8**	WD-hAECs, A549, SAEs, BEAS-2B, Hep-2 [6,7,9,13,15,16,17,20,21,22,23,24,25,27,28,30,36,42,56,57,60,61,64,66,67]	Mainly involved in neutrophil trafficking [58]	Recruits neutrophils via CXCR1 and CXCR2 signaling [58]	2 [16,28,66] 6 [16,28,64,66] 12 [16,23,61] 24 [15,16,17,20,24,27,28,36,42,58,61,66] 48 [9,13,15,22,23,24,30,42,61,64] 72 [24,42] 96 [6,7,20,21,22,60] 120 [7] 144 [7]
**CXCL9/MIG**	WD-hAECs, SAEs [9,17]	Promotes T-cell and NK cell trafficking; associated with T_h_1 responses [58]	Recruits T-cells and NK cells via CXCR3 signaling [58]	24 [17] 48 [9]
**CXCL10/IP-10**	WD-hAECs, A549, SAEs, BEAS-2B [6,7,9,13,16,17,21,68]	Promotes T-cell and NK cell trafficking; associated with T_h_1 responses [58]	Recruits T-cells and NK cells via CXCR3 signaling [58]	18 [16] 24 [7,17,68] 48 [9,13,68] 72 [13,68] 96 [6,7,21] 120 [7] 144 [7]
**CXCL11/I-TAC/IP-9**	WD-hAECs, A549 and SAEs [6,9,57]	Promotes T-cell and NK cell trafficking; associated with T_h_1 responses [58]	Recruits T-cells and NK cells via CXCR3 signaling [58]	48 [9] 96 [6]
**HMGB1**	hAECs and A549 [36,50]	Pleiotropic cytokine, initiates self-perpetuating inflammatory responses; known pyrogen [69]	Induces the secretion of cytokines and chemokines from tissue cells and lymphocytes via TLR signaling [69]	24 [36]
